# Molecular docking analysis with IC_50_ Assay FAK against for FDA approved drugs

**DOI:** 10.6026/973206300213165

**Published:** 2025-09-30

**Authors:** Zeel Gandhi, Dale D. Tang, Ajay Nayak, Jitendra D. Belani

**Affiliations:** 1 Department of Pharmaceutical Sciences, College of Pharmacy, Thomas Jefferson University, Philadelphia, PA 19107; 2 Department of Molecular Cellular Physiology, Albany Medical College, Albany, New York; 3 Department of Translational Research, Sidney Kimmel Medical College, Thomas Jefferson University, Philadelphia, PA 19107

**Keywords:** Focal Adhesion Kinase, drug repurposing, FDA-approved drugs, molecular docking, GOLD, PyRx, IC_50_ assay, Ponatinib

## Abstract

Focal Adhesion Kinase (FAK) plays a key role in cancer progression, making it a promising drug target. Hence, we screened 2,000 FDA-approved drugs using two docking tools, PyRx and GOLD. Top hits were analyzed for predicted binding affinities and molecular interactions, and selected compounds were tested in a luminescence-based IC_50_ assay. Ponatinib emerged as a promising FAK inhibitor, showing favorable binding and measurable activity.

## Background:

Focal Adhesion Kinase (FAK) is a non-receptor tyrosine kinase that is important in regulating cell adhesion, migration, proliferation, and survival [1]. Its aberrant activation is strongly associated with tumor progression and metastasis in several malignancies, including breast, ovarian, prostate and pancreatic cancers [2]. Overexpression of FAK has been correlated with poor prognosis, increased invasiveness, and resistance to therapy [3]. As such, FAK represents an attractive therapeutic target in oncology. Despite its biological relevance, no FAK inhibitor has yet received regulatory approval. Several small-molecule inhibitors, such as Defactinib and GSK2256098, are currently under investigation in preclinical or clinical trials, but the development pipeline remains limited [4]. Furthermore, many of these compounds function as ATP-competitive inhibitors, often facing specificity, toxicity, or pharmacokinetics challenges [5]. Drug repurposing, which helps identify new therapeutic uses for existing drugs, has emerged as a promising strategy for accelerating drug discovery [6]. This approach bypasses much of the early-phase development timeline by leveraging existing safety and pharmacokinetic data [7]. Drug repurposing is also particularly appealing given the many FDA-approved compounds that act on related pathways in the context of kinase-targeted therapy [8]. In recent years, computational tools such as molecular docking have been increasingly employed to predict drug-target interactions and prioritize candidates for repurposing [9]. This study aimed to evaluate whether existing FDA-approved drugs could serve as FAK inhibitors by binding to their kinase domains. We employed an *in-silico* screening approach using two docking platforms (PyRx and GOLD) to estimate binding affinity and explore molecular interactions with the FAK active site [10, 11- 12]. Top candidates were then subjected to an *in vitro* FAK kinase assay to determine their inhibitory potency as reflected by IC_50_ values. Therefore, it is of interest to report FDA-approved compounds with potential FAK inhibitory activity identified via *in silico* and *in vitro* analyses.

## Materials and Methods:

This study followed a two-stage screening approach that combined virtual screening of FDA-approved drugs with experimental validation through an *in vitro* FAK kinase inhibition assay. As outlined in Figure 1 (see PDF), we first used molecular docking tools to predict how well each compound might bind to the active site of Focal Adhesion Kinase (FAK). Compounds with favorable docking scores and meaningful interaction profiles were shortlisted for follow-up testing. An ATP-based luminescence assay was then used to measure the ability of selected compounds to inhibit FAK activity *in vitro*. The complete workflow, including ligand preparation, docking, interaction analysis, and biological validation, is summarized below.

## Ligand library preparation:

A library of approximately 2,000 FDA-approved drugs was retrieved from DrugBank [13] in SMILES format. Ligands were converted into three-dimensional structures and energy-minimized using CHARMM-GUI Ligand Reader & Modeler as shown in Figure 2 (see PDF) [14]. The resulting PDB files were processed in PyRx 0.9.8, where Gasteiger charges were added and torsions were defined using the integrated Open Babel utility.

## Protein preparation:

The three-dimensional crystal structure of human Focal Adhesion Kinase (FAK) was downloaded from the Protein Data Bank (PDB ID: 2IJM) [15]. The protein was prepared using BIOVIA Discovery Studio Visualizer 2020 by removing heteroatoms, water molecules, and co-crystallized ligands (Dassault) [16]. Hydrogen atoms were added, and the structure was saved in PDB format as illustrated in Figure 3 (see PDF). The binding pocket was predicted using FpocketWeb [17] and cross-referenced with literature to define the ATP-binding region for docking.

## Molecular docking:

Two docking platforms were used to screen the drug library:

[1] PyRx 0.9.8 (Autodock Vina Engine): A grid box was defined to include the ATP-binding site of FAK. Docking parameters were kept at the default. Binding affinities were reported as ΔG values (kcal/mol), and the top-scoring pose for each compound was selected.

[2] GOLD (Optimization for Ligand Docking CCDC): Docking was performed using the same binding site coordinates. Each ligand was evaluated using the GoldScore ChemPLP scoring function. All compounds were ranked, and reproducibility was confirmed across docking runs. Binding affinities are reported as negative ΔG values (kcal/mol) from PyRx and arbitrary GoldScore units from GOLD. PF-573228, a known FAK inhibitor, was used as a control. Ponatinib emerged as one of the top candidates across both platforms as shown in Table 1 (see PDF).

## Interaction analysis:

Ligand-protein complexes were visualized and analyzed using BIOVIA Discovery Studio Visualizer. Key interactions, including hydrogen bonds, hydrophobic contacts, and pi-stacking, were identified for the top-ranked compounds. Two-dimensional interaction diagrams were also generated using the same platform. Figure 4 (see PDF) compares the key interactions of Ponatinib and PF573228 within the FAK active site, highlighting hydrogen bonds, hydrophobic contacts, and π-π stacking interactions that contribute to binding.

## FAK Kinase inhibition assay:

We tested the top docking hits for their ability to inhibit FAK kinase activity using a luminescence-based assay (Kinase-Glo Max, BPS Bioscience, Cat# 40722) [18]. Compounds were evaluated over a concentration range from 0.1 nM to 100 µM. Luminescence was used to quantify residual ATP using the Promega GloMax® Multidetection System. IC_50_ values were calculated in GraphPad Prism 9.5 using nonlinear regression (log inhibitor vs. response). Ponatinib (compound 51) and PF-573228 (compound 53, positive control) showed strong inhibition with IC_50_ values of 32 µM and 7.5 nM, respectively. Other compounds showed minimal activity. Data are expressed as percentage FAK activity relative to the untreated control as displayed in Figure 5 (see PDF).

## Discussion:

This study evaluated whether FDA-approved drugs could be repurposed to inhibit Focal Adhesion Kinase (FAK), an important player in cancer progression. Using a two-step approach that included computational docking followed by biochemical testing, we identified Ponatinib as a promising hit. Docking with both PyRx and GOLD helped us prioritize compounds with high predicted binding affinity and strong interactions with key FAK residues, including Leu530, Cys611, and Arg545. GOLD produced more consistent scoring, allowing us to rank compounds with better confidence. Ponatinib, originally developed as a multi-kinase inhibitor, showed favorable docking scores and measurable inhibition of FAK *in vitro*. Although its IC_50_ value was higher than that of the positive control, the result supports its potential as a secondary FAK inhibitor. These findings suggest that drug repurposing can uncover new targets for existing compounds. However, not all top-scoring compounds from docking translated into experimental inhibition, highlighting a known limitation of virtual screening. Predicted affinity doesn't always reflect biological activity due to factors like solubility, off-target effects, or limited access to the active site under assay conditions. Despite the preliminary nature of the study, this combined screening approach proved useful for narrowing down candidates. Incorporating cell-based assays or additional selectivity testing in future studies could strengthen the evidence for repurposed FAK inhibitors.

## Conclusion:

We explored whether FDA-approved drugs could inhibit FAK through a combination of molecular docking and biochemical validation. Ponatinib emerged as a lead candidate, showing both strong predicted binding and measurable *in vitro* inhibition. These results support a practical, cost-effective approach for early-stage drug repurposing. While further studies are needed to confirm therapeutic relevance, this workflow offers a useful foundation for identifying and validating kinase inhibitors using widely accessible tools and resources. Thus, we show may guide future efforts to optimize kinase inhibitors through accessible, hybrid screening workflows.

## Funding statement:

This research received no external funding. There are no relevant financial or non-financial competing interests to report.

## References

[1] Zhang C, Rao Y (2025). ACS Med Chem Lett..

[2] Hu HH (2024). Front Pharmacol..

[3] Yang M (2024). Biochem Pharmacol..

[4] Zhu SK (2025). RSC Adv..

[5] Shyam S (2023). Signal Transduct Target Ther..

[6] Pushpakom S (2019). Nat Rev Drug Discov..

[7] Bhatia T, Sharma S. (2025). Curr Med Chem..

[8] Knapp S (2018). Br J Cancer..

[9] Gan JH (2023). Acta Pharmacol Sin..

[10] Trott O, Olson A.J. (2010). Journal of Computational Chemistry..

[11] Dallakyan S, Olson A.J. (2015). Methods Mol Biol..

[12] Verdonk M.L (2003). Proteins..

[13] https://go.drugbank.com/.

[14] https://www.charmm-gui.org/.

[15] https://www.rcsb.org/.

[16] https://www.3ds.com/.

[17] https://durrantlab.pitt.edu/.

[18] Jiang H (2016). Nat Med..

